# Moho Imaging with Fiber Borehole Strainmeters Based on Ambient Noise Autocorrelation

**DOI:** 10.3390/s24134252

**Published:** 2024-06-30

**Authors:** Guoheng Qi, Wenzhu Huang, Xinpeng Pan, Wentao Zhang, Guanxin Zhang

**Affiliations:** 1 State Key Laboratory of Transducer Technology, Institute of Semiconductors, Chinese Academy of Sciences, Beijing 100083, China; qigh@semi.ac.cn (G.Q.); hwzhu@semi.ac.cn (W.H.); 2 Center of Materials Science and Optoelectronic Engineering, University of Chinese Academy of Sciences, Beijing 100049, China; 3 Hunan Key Laboratory of Nonferrous Resources and Geological Hazards Exploration, Central South University, Changsha 410083, China; panxinpeng@csu.edu.cn; 4 School of Geosciences and Info–Physics, Central South University, Changsha 410083, China; 5 Optoelectronic System Laboratory, Institute of Semiconductors, Chinese Academy of Sciences, Beijing 100083, China; zhangguanxin@semi.ac.cn; 6 College of Materials Science and Opto-Electronic Technology, University of Chinese Academy of Sciences, Beijing 100049, China

**Keywords:** Moho imaging, fiber sensors, borehole strainmeters, ambient noise, seismic interference

## Abstract

Moho tomography is important for studying the deep Earth structure and geodynamics, and fiber borehole strainmeters are broadband, low-noise, and attractive tools for seismic observation. Recently, many studies have shown that fiber optic seismic sensors can be used for subsurface structure imaging based on ambient noise cross-correlation, similar to conventional geophones. However, this array-dependent cross-correlation method is not suitable for fiber borehole strainmeters. Here, we developed a Moho imaging scheme for the characteristics of fiber borehole strainmeters based on ambient noise autocorrelation. S-wave reflection signals were extracted from the ambient noise through a series of processing steps, including phase autocorrelation (PAC), phase-weighted stacking (PWS), etc. Subsequently, the time-to-depth conversion crustal thickness beneath the station was calculated. We applied our scheme to continuous four-component recordings from four fiber borehole strainmeters in Lu’an, Anhui Province, China. The obtained Moho depth was consistent with the previous research results. Our work shows that this method is suitable for Moho imaging with fiber borehole strainmeters without relying on the number of stations.

## 1. Introduction

The Moho discontinuity is one of the most important discontinuities on Earth, and obtaining its depth is highly important for studies of the crust–mantle structure and geodynamics [1,2]. The density contrast near the discontinuity usually manifests as a change in the velocity of seismic waves, so seismic tomography is a widely used method for Moho imaging [3,4].

Recently, fiber borehole strainmeters have become popular for seismic observation because of their advantages, which include a wide frequency band, low self-noise, and resistance to extreme environments [5]. Many studies have shown that, by cross-correlating the ambient noise continuously recorded by fiber optic seismic sensors, a coherent surface wave (e.g., Rayleigh wave or Love wave) can be retrieved that travels from one receiver to the other without using active seismic sources (e.g., explosives) or earthquakes [6,7,8]. Then, by extracting the dispersion curves or using direct inversion methods, fiber optic sensors can provide the same underground structural information that conventional sensors (e.g., geophones) provide [9]. However, this ambient noise cross-correlation method, which is commonly used for distributed acoustic sensing (DAS) and nodal fiber optic seismometer arrays, relies on an observation array with a scale corresponding to the detection depth and is not suitable for fiber borehole strainmeters without scale.

In contrast, the ambient noise autocorrelation method is independent of the size of the arrays and is becoming an attractive tool for obtaining Moho and lithosphere–asthenosphere boundary (LAB) images with a single station [10,11,12,13]. In 1968, Claerbout showed that the autocorrelation of transmitted waves was equal to the reflection response of these waves through a layered medium [14]. Subsequent studies extracted the P-wave reflection response from vertical component noise autocorrelations and calculated the depth of the discontinuities from a single station [3,10,11,12,15,16].

Recent studies have shown that the results from horizontal component noise autocorrelation are consistent with those from vertical component autocorrelation or the receiver function (RF) method [13], which means that ambient noise autocorrelation is a suitable and promising method for use with fiber borehole strainmeters because the number of fiber strainmeters is often low and only horizontal directions are observed. However, to our knowledge, this method has not been developed for fiber borehole strainmeters in Moho imaging. 

**Figure 1:**
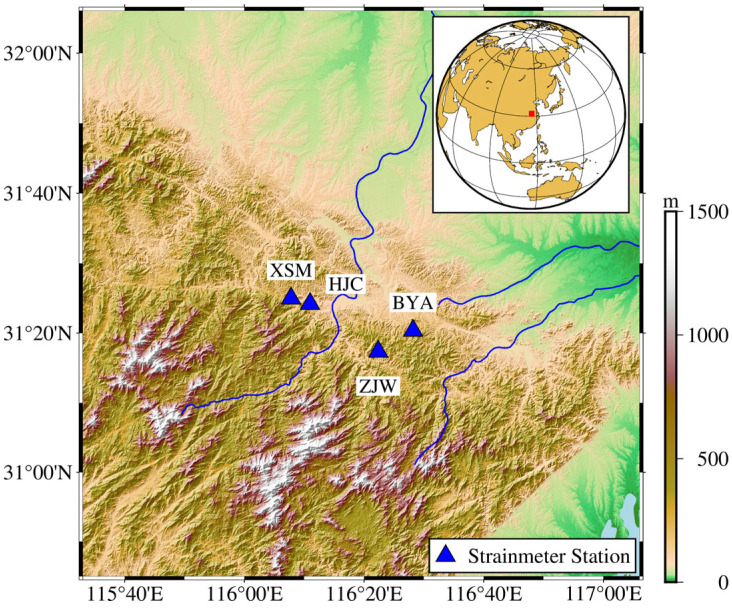
Geographic location and map overview.

In this study, we investigated the utility of fiber borehole strainmeters for Moho imaging with ambient noise autocorrelation. The data processing program mainly includes three parts: preprocessing, autocorrelation, and Moho imaging. Using ambient noise recorded by fiber strainmeters located in Lu’an, Anhui Province, China (Figure 1), we successfully retrieved S-wave reflections from the Moho that were suitable for Moho imaging, and our final results were in good agreement with our previous work. Finally, we discussed the sensitivity of the method and the uncertainty of the results.

## 2. Data and Methods

### 2.1. Data

We used data from four 4-component borehole fiber strainmeters located in Lu’an, Anhui Province, China (Figure 1). The research area was located on the northern margin of the Dabie Orogen belt, which is the contact zone between North China and the Yangtze plate, where seismic activity is concentrated [17]. Previous studies have shown that the Moho depth in this area is 33–36 km [18,19]. 

The observation equipment developed by the Institute of Semiconductors, Chinese Academy of Sciences [20] was placed 60 m underground in a drilling well and coupled to the surrounding rock mass with cement. The system comprises strain sensors, demodulators, and real-time waveform display units. These sensors are based on fiber Michelson interferometers. The laser is sent down the well through the optical fiber, divided into two beams via a 2 × 2 coupler, and then enters the two optical sensing fibers, one of which is coupled to the surrounding rock. When there is a disturbance in the external environment, the phase difference in the beams in the two sensing fibers will change. By demodulating the change in the phase of the interference fringes, then the external strain disturbance can be measured. The demodulator uses Phase Generating Carrier algorithm based on arctangent function (PGC-ARCTAN) to convert the phase difference changes of interferometers into strain signals. Four fiber optic sensors are integrated into the cylindrical probe. They are on the same horizontal plane and have an angle of 45 degrees with each other. More detailed description of this fiber borehole strainmeter’s principle can be found in [20]. Compared with traditional equipment, optical fiber instruments are not easily disturbed by electromagnetic fields and can still work stably in high-temperature and high-pressure environments.

When an external disturbance occurs, the refractive index of the strain-sensitive fiber changes, thereby changing the optical phase characteristics of the laser. Acceleration and strain signals can be obtained by demodulating the optical phase difference change through a demodulator. 

We used strain observation data for the whole year 2023 (there may be missing data due to power outages and equipment restarts).

### 2.2. Methods

**Figure 2:**
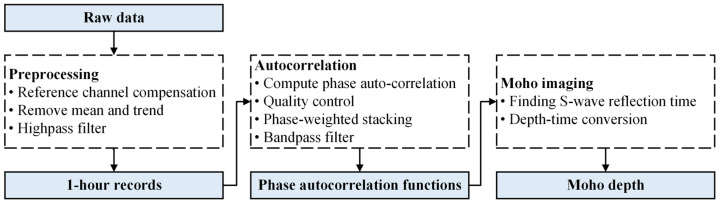
Workflow for obtaining Moho depth with single-fiber borehole strainmeters. Four main steps in this research are preprocessing, autocorrelation, quality control, and Moho imaging.

**Figure 3:**
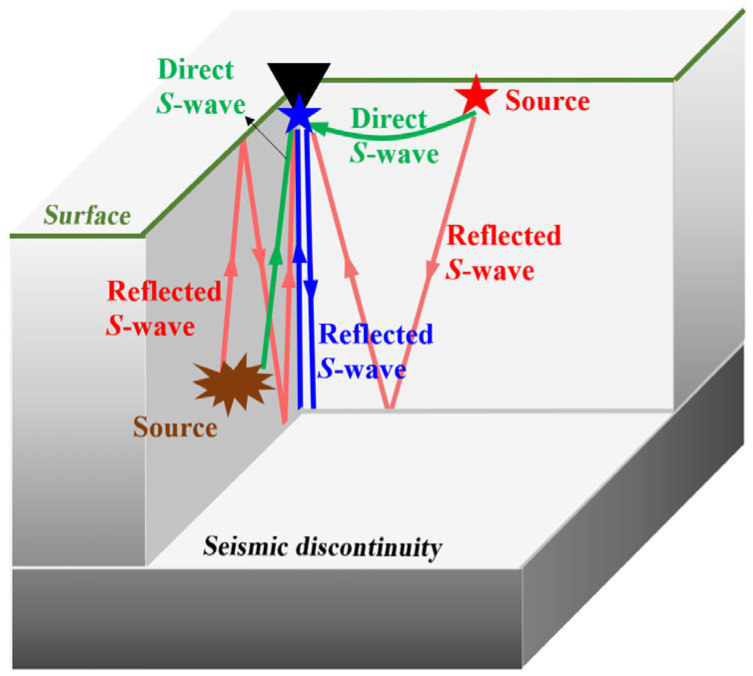
Basic principle of ambient noise autocorrelation. The data recorded by a 4-component strainmeter (black triangle) include reflected S-wave (red arrows) and direct S-wave (green arrows) from nearby noise sources at the surface (red star) or from distant sources (e.g., teleseismic sources) (explosive shape). A stacked ACF is equivalent to an observation record (blue arrow) that is excited at the observation point (blue star) and received at the same location. Adapted from Gómez-García, Lebedev, Meier, Xu, Le Pape, and Wiesenberg [15] and Schuster [22].

By considering data characteristics, we designed a workflow shown in Figure 2 to obtain high-quality Moho depth based on ambient noise autocorrelations (the principle is shown in Figure 3).

#### 2.2.1. Data Preprocessing

Before ambient noise autocorrelation, we preprocessed the raw observation data to remove instrument responses and earthquake signals. The preprocessing procedure, described in Figure 3, was adapted from Bensen, et al. [21]. 

First, we down-sampled the row data to 10 Hz because the noise band used to extract the body wave is usually less than 10 Hz. The data were then cut into 1-h time windows, and nonlinear trends were removed. We used the observation data minus the reference channel data to eliminate the instrument’s response and then used spline function fitting to remove the residual nonlinear trend. In contrast to previous studies, the phase autocorrelation method adopted in this paper was not affected by strong amplitude transient signals, such as earthquakes, so time-domain normalization and spectral whitening were not necessary during preprocessing. Finally, a Butterworth high-pass filter with a frequency of 0.5 Hz was applied for each time window, similar to [13]. This lower high-pass cutoff frequency in the preprocessing steps can ensure we will not miss any potentially effective signals.

#### 2.2.2. Phase Autocorrelation and Stacking

After preprocessing, we calculated each time window’s phase autocorrelation (PAC) functions. This method originates from phase cross-correlation (PCC) [23], which is based on the coherence of the instantaneous phase of two signals and is not easily affected by instantaneous strong amplitude signals. This method was applied to vertical and horizontal seismic noise cross-correlation [13]. For a discrete random signal *u*(*t*), phase autocorrelation can be calculated according to Schimmel [23].

(1) $$c_{pac}\left(t\right)=\frac{1}{2N}\sum_{\tau =\tau_{0}}^{\tau_{0}+T}\left\{{\left|e^{i\Phi (t+\tau )}+e^{i\Phi (\tau )}\right|}^{\eta }-{\left|e^{i\Phi (t+\tau )}-e^{i\Phi (\tau )}\right|}^{\eta }\right\},$$

Here, *c_pac_*(*τ*) represents the phase cross-correlation function, *τ* represents the delay time, *N* represents the number of sampling points of the signal in the time interval [*t*_0_, *t*_0_ + *T*], the sensitivity of phase cross-correlation can be changed by the value of *η*, and Φ(*t*) is the instantaneous phase of the observation signal. This may lead to an improved signal-to-noise ratio (SNR), while absolute correlation values may decrease [24], but also carries with it the risk of deformities in the signal waveforms. Here, we set *η* = 1, similar to [23,24]. The instantaneous phase Φ(*t*) of the analytic signal can be calculated by the Hilbert transform.

Then, all PAC functions were stacked via a nonlinear stacking method, phaseweighted stacking (PWS) [25]. The phase stack was calculated by normalizing the instantaneous phase of the analytic signal, which was between 0 and 1, with *N* being the number of traces involved in the stack.

(2) $$c_{ps}\left(t\right)={\left|\frac{1}{N}\sum_{j=1}^{N}e^{{i\Phi }_{j}(t)}\right|}^{\eta },$$

Then, the PWS results *g_pws_*(*t*) were obtained by multiplying the phase stack *c_ps_*(*t*) and the linear stack results *g_ls_*(*t*).

(3) $$g_{pws}\left(t\right)=g_{ls}\left(t\right)c_{ps}\left(t\right)=\frac{1}{N}\sum_{i=1}^{N}c_{pac}^{i}(t)\frac{1}{N}{\left|\sum_{j=1}^{N}e^{{i\Phi }_{j}(t)}\right|}^{\eta },$$

**Figure 4:**
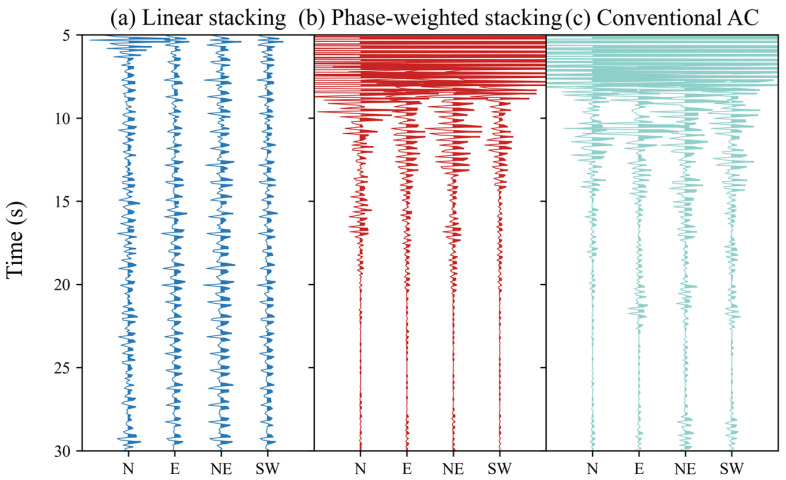
Comparison between the linear stacking (**a**), PWS (**b**) of PAC, and PWS (**c**) of the conventional autocorrelation (AC) for Station HJC.

A comparison was made between the linear stacking and phase-weighted stacking results of one-year-long phase autocorrelation. Figure 4 shows noticeable signal-to-noise ratio (SNR) differences between the stack methods. Therefore, the PWS method was used in this study. We also conducted a comparative analysis using traditional autocorrelation techniques. Our findings indicate that the autocorrelation waveforms obtained using both methods are generally similar, which supports the reliability of our approach. Additionally, we observed that the phase autocorrelation method yields waveforms with higher consistency across different components at the same station than traditional autocorrelation. This improved consistency may reflect the advantage of our method in noise suppression.

**Figure 5:**
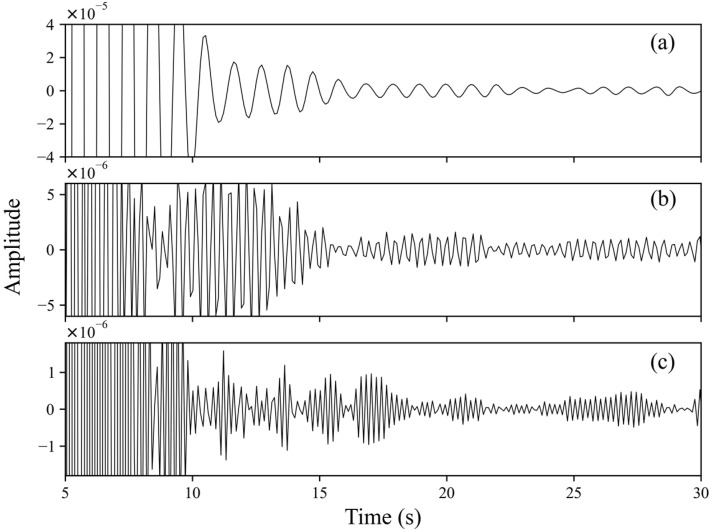
The results of bandpass filtering were applied to PAC in different frequency bands for Station BYA. Bands are (**a**) 1–2 Hz, (**b**) 2–4 Hz, and (**c**) 4–5 Hz.

After stacking, the results were bandpass-filtered from 2 to 4 Hz. Figure 5 compares the results of filtering in different frequency bands: the period of the autocorrelation function in the 1–2 Hz is large, and the resolution is low, making it difficult to identify S-wave reflection signals, while there are many wave packets in the 4–5 Hz range, which results in difficulty in identifying the S-wave reflection signal. In contrast, the 2–4 Hz results are slightly better, and signs of SmS signals can be seen in the phase autocorrelation function.

#### 2.2.3. Time-Deep Conversion

**Figure 6:**
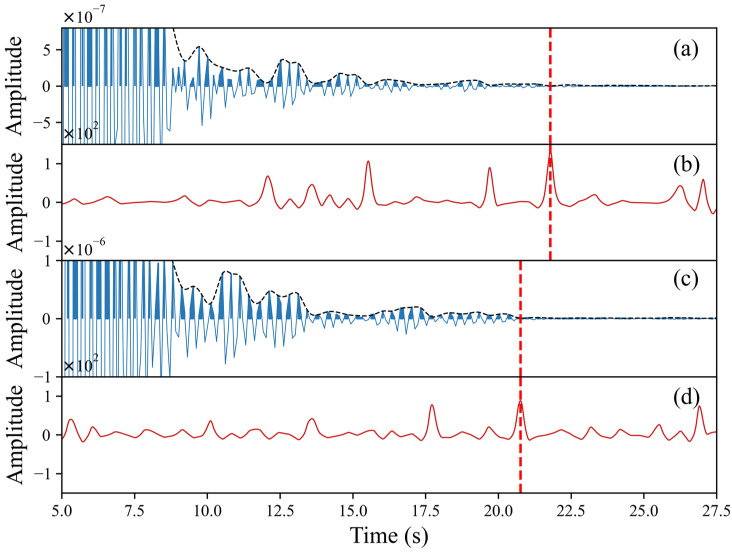
The phase autocorrelation waveform (solid black line) and its envelope (dotted black line) for Stations XSM (**a**) and HJC (**c**) and their second-order derivative of the envelope (**b**,**d**). The red vertical dotted line is the S-wave reflection time.

Previously, scholars directly identified the reflection response for Earth’s interior from the peaks of autocorrelation waveforms. However, this method was highly subjective, and, in some areas with gradient Moho surfaces, there may not be an obvious reflection response [11]. Therefore, we used automatic methods to measure reflectivity changes to determine the velocity discontinuity depth [11,12,13]. The main steps of the depth picking algorithm for velocity discontinuity were as follows: (1) we calculated the envelope of all phase autocorrelation waveforms superimposed during the research period at each station, (2) we calculated the second-order derivative of the envelope and weighed it with PAC’s amplitude, and (3) we selected the local maximum value of the weighted second-order derivative in the time window provided by prior information and identified the position of the velocity discontinuity. Figure 6 shows two examples for Stations XSM and HJC.

## 3. Results

Using our proposed procedure (see Figure 2), we calculated the phase autocorrelations hourly at four stations, XSM, HJC, BYA, and ZJW, and the stacked autocorrelations for each hour. After a zero-lag time, a large pulse lasted approximately 7 s because of a strong response near the surface (see Figure 6a,c). The details of the waveform between 7 and 30 s usually varied daily, and it was difficult to observe consistent features with time. Therefore, it may be necessary to improve the SNR by increasing the length of the data in the stack and adopting stack methods. In this study, we used PWS to improve the SNR and reduce the dependence on the amount of data.

**Figure 7:**
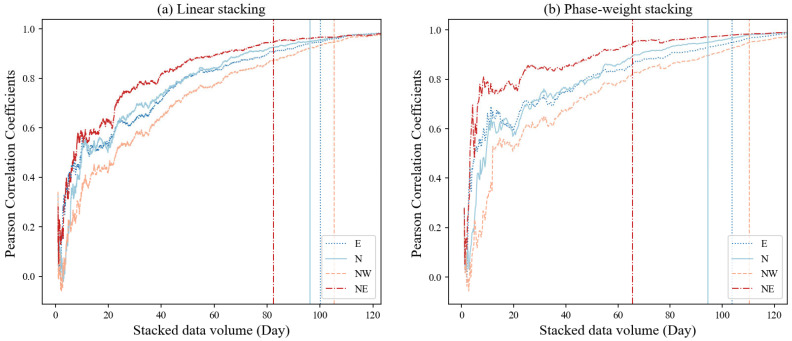
Pearson correlation coefficients between the total linearly stacked ACF and the stacking of hourly ACFs as a function of increasing stacked hours, from 1 to 3000 h (approximately 125 days, which is also the total number of stacked days), for the E, N, NW, and NE components of Station XSM. The vertical lines indicate the threshold after which the correlation coefficient permanently increases to above 0.9. Only autocorrelations for the lag time window of 5–30 s were used to calculate the correlation coefficients.

To achieve the amount of stacked data needed to obtain a stable result, we continuously stacked the hourly autocorrelations and calculated the Pearson correlation coefficients between the total linearly stacked ACF and an increasing number of stacked hourly ACFs (from 1 to 3000 h, approximately 125 days), similar to Gómez-García, Lebedev, Meier, Xu, Le Pape, and Wiesenberg [15]. We assumed that, once a Pearson correlation coefficient permanently increased above 0.95, the autocorrelation was stable and would not change as the amount of data increased. For most stations, the coefficient was above 0.95 when there were greater than 4 months of data. Figure 7 shows the Pearson correlation coefficients for Station XSM, which generally increase as the number of stacked hours increases. However, it must be noted that the correlation coefficient may decrease or fluctuate with the addition of new signals when the amount of data is lower, as shown in Figure 7. In addition, we also found that the time thresholds required for different components at the same station are also different, which may be related to the performance differences of the sensors themselves. 

**Figure 8:**
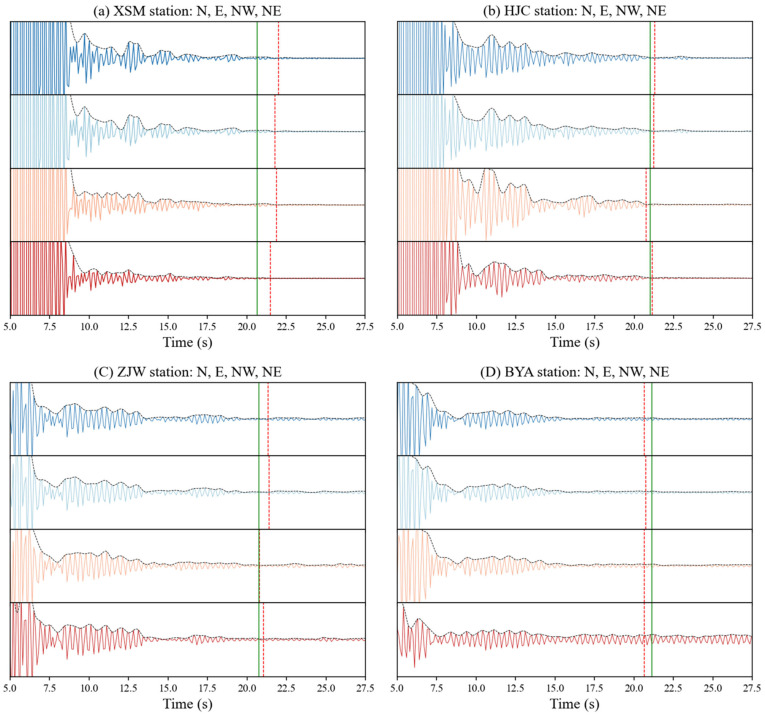
Autocorrelations and their 2nd derivatives for the 4-component fiber strainmeters XSM, HJC, ZJW, and BYA. The red vertical dotted lines are the S-wave reflection time computed in this study, and the green lines are that time according to [29].

Analyzing the consistency across the different components allowed us to evaluate the health of the fiber strainmeters and the accuracy of the results. We calculated the results for four horizontal components at all the stations. As shown in Figure 8, the ACFs for the same station’s E, N, NW, and NE components were similar, demonstrating the high degree of self-consistency for the ACFs. Due to location differences, there were small differences in the ACFs for different stations.

The average S-wave velocities in the crust below these stations are assumed to be the same because they are very close together (the maximum distance between two stations is approximately 30 km), and this velocity ${\bar{v}}_{s}$ was calculated to be 3.568 km/s according to the CRUST 1.0 model [26]. As shown in Table 1, the Moho depths after the time-to-depth conversion were very close to the interpolation results according to the CRUST 1.0 model, with a maximum difference of 2.02 km and a minimum difference of 0.01 km, reflecting the proposed algorithm’s reliability. In addition, our study’s findings are consistent with these previous results, including the average depth of approximately 34 km by the P-wave receiver function H–κ method [27], the estimation of a 35–40 km range that used the 3D inversion of magnetotelluric array data [28], and a 35 km-thick crustal cross section of the Dabie Shan orogenic belt, in east central China, based on a 400 km-long seismic refraction profile [18].

## 4. Discussion

Our study demonstrates that the Moho depth can be obtained via ambient noise autocorrelation with fiber strainmeters. Our methodology includes three steps: preprocessing, autocorrelation and stacking, and time-depth conversion.

The design and range of bandpass filtering play key roles in the preprocessing step. A Butterworth filter is commonly used because it does not cause phase delay [12,13]. The frequency band depends on the dataset used and varies from 1–6 s [15] to 2–4 Hz [11], with a variety of ranges in between [12,13,30]. A frequency band that is too low is not suitable because the sidelobes of the main peak may extend more than the reflection time [12]. If the frequency band is too high, a change in reflectivity is often not visible for most broadband stations but is sometimes detectable for short-period stations [11,12]. After comparing different frequency bands, 2–4 Hz was chosen for our analysis, similar to [11].

Less noise can be obtained in the final stacked results by choosing a suitable type of stack or quality screening [21]; for example, time–frequency or time–scale domain phase–weighted stacking (tf/ts–PWS) may be chosen to improve the convergence of empirical Green’s functions (EGFs) when extracting the surface waves from ambient noise [31]. The application of a PWS here improves the SNR of the final autocorrelation (see Figure 4) because it can enhance the coherent phases [23,32]. When the amount of data is small, the nonlinear stacking method can quickly improve the stability of the superposition results, as compared in Figure 7. When the amount of data is large, although the linear stacking results will tend to be stable, the signal-to-noise ratio will not increase with the increase in the amount of data. This may mean that the data volume cannot replace the advanced stacking algorithms in improving the SNR.

Instead of the SNR [33] or root-mean-square (RMS) value [12,13], we used Pearson correlation coefficients to estimate the amount of stacked data when the results were stable because these coefficients describe the direction and strength of the linear correlation between the traces [15]. However, we found that the correlation was easily influenced by signal windows with large amplitudes, such as high pulses after zero lag; therefore, we chose a signal window of 5–30 s to calculate the correlation coefficient. We achieved stable results using four-month-long datasets (less than the 259 days of Becker and Knapmeyer-Endrun [13] and the 140 days of Gómez–García et al. (2023). We speculate that this is related to the low-noise performance of the fiber strainmeters and 60-m-deep well observation environment.

**Figure 9:**
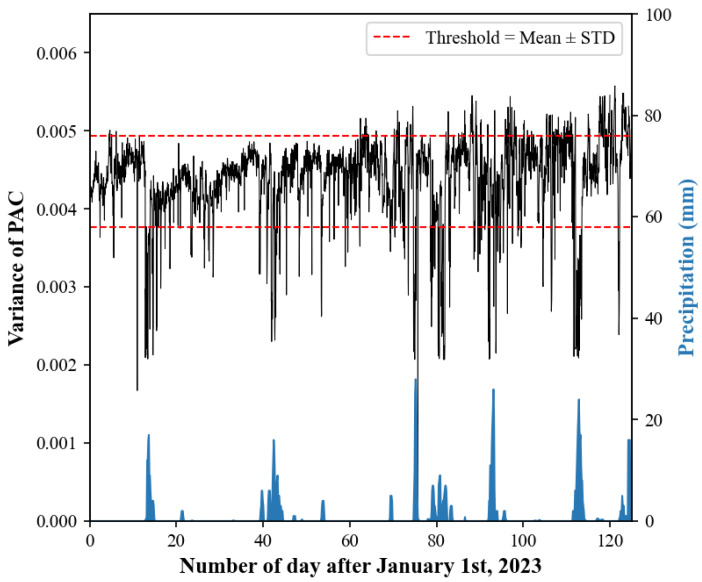
Variance of autocorrelation waveforms and local precipitation for Station XSM.

To explore the relationship between the stacking results and raw data quality, we calculated the variance of the PAC corresponding to each time window. Figure 9 shows the variances for Station XSM over time. The general amplitudes maintained relative stability, and we did not find the obvious seasonality characteristics found by other scholars [12,13]. However, on some days, the variance became suddenly and noticeably smaller, and these periods of sudden variance changes correspond to the occasional drop in the correlation coefficient curve (see Figure 7). This may have been due to greater natural or man-made interference, such as human activities, changes in the water table, weather, etc. For some stations, precipitation seems to have a certain relationship with data quality (see Figure 9). When precipitation occurs, the variances in the PACs will decrease; when this aspect of the PACs is superimposed, the Pearson correlation coefficient curve will fluctuate accordingly. So, we speculate that the larger surface vibration noise generated by local precipitation or wind may interfere with the imaging of the deep Earth. For this situation, we suggest taking quality control measures, such as setting a dual threshold to filter out PACs with obviously abnormal variances. In our study, only PACs with variances falling within the range [*v_d_*, *v_u_*] are considered for stacking, where *μ* is the mean of the PAC’s variances and *σ* is the standard deviation of the variances. This dual-threshold approach ensures that the data segments used in the analysis are of higher quality and less affected by transient noise. We reprocessed the data with these new quality control measures in place. The results show that the influence of the ambient noise factor is reduced, and fewer data are required to ensure the stability of the results (Table 1).

(4) $$v_{u}=\mu +\sigma ,$$

(5) $$v_{d}=\mu -\sigma ,$$

**Table 1:** The impact of quality control measures on the need for data volumes.

	Amount of Data Required for PAC Stabilization (Days)			
Station	XSM-E	XSM-N	XSM-NW	XSM-NE
Without quality control measures	103.75	94.50	110.50	65.50
Applying quality control measures	85.00	75.00	90.75	60.00

The determination of the Moho reflection was complex and often informed by prior information [11,34], consistency among arrays [29,34], forward modelling [34], changes in reflectivity [12,13], and even other methods [4,16,35]. In this study, we selected SmS, which is indicated by a change in reflectivity. However, there was more than one maximum point in the second derivative curve (Figure 6b,d), demonstrating this method’s shortcomings. Therefore, choosing the right target uncertainty window may be useful based on prior information. Here, we need a Moho reflection time range as a reference according to the prior Moho depth and average S-wave velocity in the previous research [19,29]. The average S-wave velocity in the crust ${\bar{v}}_{s}$ is an important factor affecting the accuracy of the final calculated Moho depth. However, accurately obtaining the average S-wave velocity in the crust at a certain location is difficult. The 2D interpolation method was used to calculate ${\bar{v}}_{s}$ according to the CRUST 1.0 model. If we assume a 5% error in ${\bar{v}}_{s}$, the reflection time range can be computed.

The final Moho depth is calculated with the reflection time and S-wave average velocity. Therefore, there are two main sources of error in this study: one is the calculation of the average S-wave velocity, which depends on the previous model, and the other is the selection of the reflection time because we found that the reflection times of different horizontal components at the same station are slightly different. The slight difference may be caused by errors in the fitted waveform envelope. We take 5% of the average speed of the S-wave as the velocity uncertainty, calculate the standard deviation of the reflection time of the four horizontal components as the uncertainty of the reflection time, and finally evaluate the uncertainty of the Moho depth according to the formula for error propagation [36] (see Table 2). Since both the S-wave velocity and reflection time contribute to this uncertainty, it is important to rely on high-quality regional velocity models, more observation data, and robust signal processing methods to mitigate these errors.

If the reflection pickup error is not considered, the phenomenon that there were differences between the results from different horizontal components is also attributed to the presence of the average anisotropy in the crust in some studies (like [13]). Previous studies have shown that the research region is located in the Qinling–Dabie Shan orogenic belt, which is believed to have formed by the northward-directed subduction of the Yangtze craton or by a microcontinent beneath the Sino-Korean craton [18,37]. The seismic anisotropy in the region may be caused by this mechanism of crustal deformation, which was previously observed by teleseismic receiver functions [38]. However, the strength of the anisotropy near the stations is not clear, and its influence on this method is subject to further evaluation. In future studies, we will develop new theories and technologies to analyze regional anisotropy’s influence.

## 5. Conclusions

In this study, with fiber borehole four-component strainmeters, we achieved high-resolution Moho imaging via ambient noise autocorrelation. The S-wave reflection time was selected by finding the change in reflectivity in the target uncertainty window and transformed into the Moho depth using the average S-wave crustal velocity. Compared with previous studies, we believe that this study has the following advantages: on the one hand, our algorithm does not depend on the number and distribution of stations, which means that only a few stations can be used to detect the crustal structure; on the other hand, we have confirmed the feasibility of using fiber optic borehole strainmeters for Moho surface imaging (as far as we know, most of the previous explorations of the deep Earth may require the use of broadband seismographs). Our results were consistent with those of the previous research. In addition, we found a difference in autocorrelation between the horizontal components. Further discussion of this phenomenon will be helpful in studying the crustal anisotropy of the region, which is one direction of our future research.

## Figures and Tables

**Table 2:** Overview of Moho depths computed from four 4-component fiber strainmeters.

Station	Moho S-Wave Reflection Time (s)	Moho Depth (km)	Moho Depth in Cheng et al. (2021) [29] (km)
XSM	21.79 ± 0.19	38.88 ± 2.82	36.86 ± 3.61
BYA	20.69 ± 0.04	36.90 ± 2.42	37.47 ± 4.14
HJC	21.11 ± 0.21	37.66 ± 2.79	37.04 ± 3.70
ZJW	21.15 ± 0.24	37.73 ± 2.86	37.72 ± 4.11

## Data Availability

Restrictions apply to the availability of the strain data. These data were obtained from China earthquake administration (CEA) and are available from CEA with permission. Details on the permission and rules of obtaining the seismic data can be found at the following website: https://data.earthquake.cn/sjgxgz/info/2016/2344.html (accessed on 23 May 2024). The precipitation data are available via the website https://rp5.ru/ (accessed on 23 May 2024). The algorithms presented in Equations (1)–(3) are available as open–source codes at https://github.com/chyiever/mohoimaging_pac (accessed on 23 May 2024).
